# Contribution of intracranial artery stenosis to white matter hyperintensities progression in elderly Chinese patients: A 3-year retrospective longitudinal study

**DOI:** 10.3389/fneur.2022.922320

**Published:** 2022-09-23

**Authors:** Tingting Zhong, Yunwen Qi, Rui Li, Huadong Zhou, Boli Ran, Jiao Wang, ZhiYou Cai

**Affiliations:** ^1^Chongqing Medical University, Chongqing, China; ^2^Department of Cardiology, Chongqing General Hospital, Chongqing, China; ^3^Department of Neurology, Chongqing General Hospital, Chongqing, China; ^4^Chongqing Key Laboratory of Neurodegenerative Diseases, Chongqing, China; ^5^Stroke Center and Department of Neurology, The First Affiliated Hospital of USTC, Division of Life Sciences and Medicine, University of Science and Technology of China, Hefei, China; ^6^Department of Neurology and Centre for Clinical Neuroscience, Daping Hospital, Third Military Medical University, Chongqing, China

**Keywords:** intracranial artery stenosis, white matter hyperintensities, magnetic resonance imaging, computed tomography angiography, retrospective longitudinal study

## Abstract

**Background and purpose:**

There have been controversial results in previous studies for the association between intracranial artery stenosis (ICAS) and white matter hyperintensities (WMHs), and the correlation of ICAS with the progression of WMHs is uncertain. The aim of this study was to investigate the association between ICAS and the progression of WMHs.

**Methods:**

In this retrospective longitudinal study, we enrolled 302 patients aged 60 years and older who had received two brain MRI scans with a 3-year interval and was examined by CTA in the first MRI scan. We measured the stenosis of major intracranial arteries by CTA and assessed the progression of WMHs using the modified Rotterdam Progression scale (mRPS). We performed binary logistic regression analyses and established linear regression model to determine the relationship between the degree of ICAS and the progression of WMHs.

**Results:**

A total of 302 patients were enrolled, of which 48.3% experienced WMHs progression. After adjustment for confounding factors, the patients with Grade 2 ICAS had an OR of 2.8 (95% CI 1.4–5.5), and those with Grade 3 ICAS had an OR of 3.0 (95% CI 1.2–7.3) for the progression of WMHs. The ICAS degree remained associated with PVWMHs but had an attenuated relation to SCWMHs. ICAS severity was significantly associated with WMHs progression scores, higher for Grade 3 ICAS [β (SE) = 0.18 (0.18)] followed by Grade 2 ICAS [β (SE) = 0.10 (0.15)] compared with Grade 1 ICAS.

**Conclusions:**

Patients with more severe ICAS are more likely to have WMHs progression and have distinct relevancy to PVWMHs and SCWMHs, which may provide clues for understanding mechanisms of WMHs progression.

## Introduction

White matter hyperintensities (WMHs) are frequently found on brain MRI of older individuals and substantially predict an increased risk of dementia, stroke, mortality, and physical disability (1, 2). Some evidence considers WMHs as small-vessel disease-related lesions (3, 4), but the exact etiology is poorly understood. Proposed mechanisms include that chronic hypoperfusion, diffuse cerebrovascular endothelial failure, and blood-brain barrier alterations (4, 5) alone or simultaneously contribute to the damage of cerebral vessels and parenchyma. Postmortem studies have shown that WMHs correlate with various degrees of demyelination, arteriolosclerosis, and mild gliosis (6). Pathologically, most WMHs lesions are ischaemic in origin.

Inefficient blood supply to brain due to luminal narrowing by atherosclerotic arterial stenosis in the upstream may be contributory to the presence and development of WMHs (7). The correlation between carotid arteriosclerosis and WMHs is still a matter of controversy, even though a large number of studies have been dedicated to identifying it (8). Most of these studies have reported no definitive association between vascular risk factors (VRFs) and WMHs (9). Intracranial- and Extracranial atherosclerosis (ICAS and ECAS) have been suggested to have different pathogeneses. Intracranial arteries might have less opportunity to be hemodynamically compensated by the circle of Willis and is related to weaker cerebral autoregulation, because they are less elastic and result in pressure-passive cerebral blood flow (10). Some cross-sectional studies have suggested that patients with ICAS, rather than ECAS, may particularly have heavier WMHs burden (7, 11, 12), while others failed to demonstrate the relevance (13, 14). Additionally, WMHs may progress insidiously rather than staying unalterable, and the risk factors for progression are uncertain. To our knowledge, only one study assessed the effects of statins on the progression of WMHs, but regrettably, it mentioned no significant correlation between ICAS and the progression of WMHs (15). Further exploration of the relationship between ICAS and WMHs is warranted.

We therefore sought to conduct the present retrospective longitudinal study in patients without acute stroke or ECAS, aiming to determine whether stenosis in major intracranial arteries could serve as a predictor for WMHs progression within a period of 3 years.

## Methods

### Study population

This study was retrospectively performed on inpatients admitted to the Department of Neurology of Daping Hospital in the city of Chongqing, China, from January 1, 2010 to December 31, 2015. Eligibility criteria included: (1) patients aged 60 years and older; (2) patients who had undergone cerebral magnetic resonance imaging (MRI) scans twice in this period with a 3-year interval; and (3) patients who had been examined by CT angiography (CTA) in the first MRI scan. Exclusion criteria were: (1) patients with a leukoencephalopathy of non-vascular origin (e.g., infectious, toxic, immunological-demyelinating); (2) patients with a diagnosis of brain tumors, acute stroke within 2 weeks, or new stroke during the 3-year interval; (3) patients with a stenosis of ≥20% in the extracranial internal carotid artery and vertebral artery (ECAS); and (4) patients without adequate clinical information. The study protocols were approved by the Institutional Review Board of the Third Military Medical University, and all patients signed written informed consent before the study started.

### Clinical assessment

The clinical data were obtained from reliable medical records, physical examinations and structured clinical interview, and consisted of demographic data, medical history, and vascular risk factors (VRFs). The demographic data collected were comprised of age and sex. Each patient's systolic and diastolic blood pressures (BP) were measured using an aneroid sphygmomanometer with a 10-min relaxing. Body mass index (BMI) was measured. Use of anti-hypertensive, antiplatelet agents, statin and anti-diabetic medications, including oral hypoglycemic agents or insulin injection, were ascertained. The VRFs included in our study were hypertension, diabetes, coronary heart disease, dyslipidaemia, previous stroke, and current smoking and alcohol habit. Individuals with hypertension were defined as those with systolic/ diastolic BP measures >140/90 mmHg in three consecutive measurements, or current use of anti-hypertensive agents, or a prior diagnosis of hypertension. Diabetes was defined as fasting blood glucose >7.0 mM or currently undergoing treatment with hypoglycemic medicine or insulin. Coronary heart disease (CHD) was determined by coronary angiography or cardiac CTA after being screened by electrocardiography or echocardiography according to the protocols for cardiac risk assessment. Dyslipidaemia was defined by a total cholesterol concentration >5.2 mM and a low-density lipoprotein cholesterol concentration >3.4 mM. Previous stroke defined by history were confirmed before a definitive diagnosis was made. Current smoking and daily alcohol use were investigated as previously described (16).

### CTA scans and determination of ICAS

CTA scanning from the aortic arch to the cranial vessels was performed on a 64-slice CT scanner (Light Speed VCT 64-slice Scanner; General Electric, Milwaukee, WI). Details of the CTA protocols have been reported previously (17). All intracranial artery stenosis in the CTA images were reviewed and calculated as percent stenosis = [(1 – (D_stenosis_/D_normal_)] × 100 (D_stenosis_ = the diameter of the artery at the site of the most severe stenosis, D_normal_ = the diameter of the proximal normal artery) (18) by two researchers (Zhong and Zhou) who were blinded to the patients' clinical information and WMHs scoring. The final stenosis percent for each vessel was defined according to the maximum value of all sites if multiple stenotic lesions were simultaneously existent. The vessels inspected include the intracranial internal carotid arteries; the proximal portions of anterior, middle, and posterior cerebral arteries (e.g., A1, M1, and P1, as well as distal segments or the communicating arteries, if present); intracranial vertebral arteries and basilar artery (19). The degree of ICAS was determined according to our previous study (19) and rated as: Grade 1 required stenosis of <20% in any vessel; Grade 3 required stenosis of ≥40% in two or more vessels; Grade 2 was assigned as intermediate lesions, which predominantly included single-vessel disease or multiple low-grade stenosis (20).

### MRI scans and WMHs grading

MRI scans were performed using a 3.0 T magnet (MAGNETOM Verio 3.0T; Siemens, Erlangen, Germany) with three high-resolution axial scans, i.e., T1- and T2-weighted and fluid attenuated inversion recovery sequences (FLAIR). The degree of WMHs severity was rated visually on axial FLAIR images by two trained investigators (Li and Cai) blinded to clinical data and ICAS assessment, using both the Age-related white matter changes (ARWMC) scale (range, 0–30) (21) and the four-class modified Fazekas scale (WMHs grade = none, mild, moderate, and severe) stratified separately for periventricular white matter hyperintensities (PVWMHs) and subcortical white matter hyperintensities (SCWMHs) (22). In brief, the ARWMC scale graded WMHs severity by giving scores 0 to 3 in bilateral five brain regions (frontal lobe, parietal-occipital lobe, temporal lobe, infratentorial region, and basal ganglia). PVWMHs were graded as: none, absent; mild, small caps or pencil-thin lining; moderate, large caps or thick smooth halo; severe, irregular PVWMHs extending into the subcortical white matter. SCWMHs were graded as: none, absent; mild, punctuate foci; moderate, beginning confluence of foci; severe, large confluent areas. Standards for Reporting Vascular Changes on Neuroimaging (STRIVE v1) (3) was consulted for identifying WMHs from other signs of cerebral small vessel disease (e.g., recent small subcortical infarcts, lacunes, prominent perivascular spaces and cerebral microbleeds).

Visual rating of WMHs progression was performed in a side-by-side fashion blinded to clinical and image details. WMH progression was rated on FLAIR images against the modified Rotterdam Progression scale (mRPS) (range, 0–9) (23), in which absence or presence of progression (0 and 1, respectively) was rated in three periventricular regions (frontal caps, occipital caps, bands), four subcortical white matter regions (frontal, parietal, occipital, temporal), basal ganglia, and infratentorial region. Progression of WMHs was defined as an increase in one point or more on the 3-year interval, which was further stratified into PVWMHs progression and SCWMHs progression with one point or more in periventricular and subcortical areas on mRPS, separately. The progression of basal ganglia and infratentorial regions was not further studied in the subgroup analysis because the sample size was too small. Disagreements over imaging assessment were resolved by discussion and consensus was reached.

### Statistical analysis

The normal distribution of the data for numerical variables was evaluated with the Shapiro–Wilk normality test or Q–Q plot test. In the univariate analyses, continuous variables which were independent and normally distributed were expressed as mean ± SD and analyzed using Student *t-*test, or were expressed as median and interquartile range and compared using the Mann–Whitney *U* test when those were not normally distributed; and χ^2^ tests were analyzed for categorical data. Binary logistic regression analyses with adjustment for age and sex and additionally for VRFs (e.g., hypertension, diabetes, CHD, dyslipidaemia, previous stroke, current smoking and daily alcohol use), and finally baseline WMHs (ARWMC scores) were performed to determine the relationship between ICAS degrees and WMHs progression. Furthermore, a linear regression model was established to describe the associations between ICAS degrees and WMHs progression scores. Then, we reapplied binary logistic regression analyses adjusting for age, gender, VRFs, and baseline PVWMHs or SCWMHs (Fazekas scores) separately to stratify the association of ICAS degrees and PVWMHs or SCWMHs progression. All *P*-values were two-tailed, and those <0.05 were considered statistically significant. The statistical analyses were performed by using SPSS22.0 for Windows (SPSS Inc., Chicago, IL, USA).

## Results

### Characteristics of the study participants

**Figure 1:**
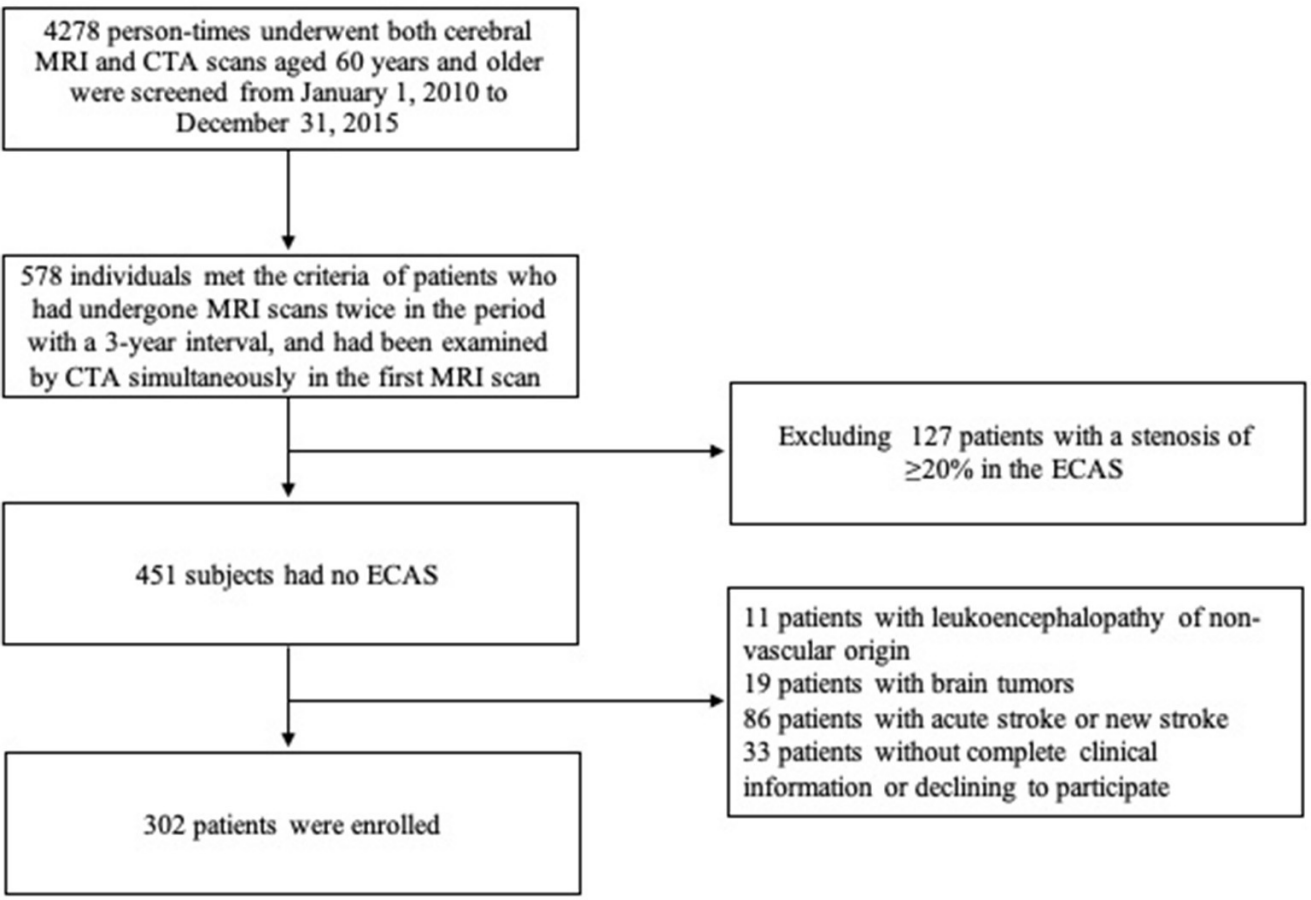
Flow chart of patient screening for the present study.

From January 1, 2010 to December 31, 2015, a total of 578 patients aged 60 years or above who met the criterion that they received two MRI scans 3 years apart and a CTA examination in their first MRI scan in our center were screened. Among them, the following patients were excluded from this study: 11 patients with leukoencephalopathy of non-vascular origin, 19 with brain tumors, 86 with acute stroke at the first MRI scan within 2 weeks or new stroke during the 3-year interval, 127 with a stenosis of ≥20% in the ECAS and vertebral artery, and 33 patients without complete clinical information or who refused to participate. Finally, a total of 302 patients (139 men and 163 women) with mean age of 69.7 ± 8.2 years were enrolled (flow chart shown in Figure 1). Amongst the enrolled patients, 186 complained of non-specific neurological symptoms (e.g., dizziness, vertigo, and numbness), 17 of headache, 5 of sleep disorders, 6 of cognitive impairment, 33 of Parkinson's disease, 20 of movement disorders, 20 of peripheral neuropathy, and 15 of other neurological conditions. Among them, 146 (48.3%) had a progression of WMHs within 3 years and showed 2.04 ± 1.14 points increase on the mRPS (one-sample *t-*test: *P* < 0.001).

Characteristics of patients with and without WMHs progression are shown in Table 1. Compared with the patients who had no WMHs progression, those who progressed were older, displayed significantly higher incidence of hypertension and diabetes, and were more likely to have a previous stroke (*P* < 0.05). The baseline WMHs score (ARWMC scale), proportion of patients who already had PVWMHs and SCWMHs (Fazekas scale) at baseline, and as well as the severity of ICAS were higher in the patients who had WMHs progression compared with those who did not progress (*P* < 0.001). Unexpected association also appeared with antihypertensive use (*P* = 0.010).

**Table 1:** Characteristics of patients with and without WMHs progression.

**Patient characteristics**	**All patients (*n* = 302)**	**WMHs**		
		**Non-progression (*n* = 156)**	**Progression (*n* = 146)**	***P***
Age (years)	69.7 ± 8.2	67.0 ± 8.5	72.6 ± 6.7	< 0.001**
Gender (male)	139 (46.0%)	68 (43.6%)	71 (48.6%)	0.380
BMI (kg/m^2^)	23.6 ± 3.4	23.4 ± 3.4	23.8 ± 3.4	0.283
Systolic BP (mmHg)	136.0 ± 18.4	134.8 ± 18.8	137.3 ± 18.0	0.236
Diastolic BP (mmHg)	76.4 ± 12.1	77.0 ± 13.0	75.7 ± 11.0	0.378
Hypertension	173 (57.3%)	73 (46.8%)	100 (68.5%)	< 0.001**
Diabetes	46 (15.2%)	15 (9.6%)	31 (21.2%)	0.005**
Coronary heart disease	84 (27.8%)	43 (27.6%)	41 (28.1%)	0.920
Dyslipidaemia	47 (15.6%)	30 (19.2%)	17 (11.6%)	0.069
Previous stroke	39 (12.9%)	12 (7.7%)	27 (18.5%)	0.005**
Current smoking	23 (7.6%)	11 (7.1%)	12 (8.2%)	0.702
Daily alcohol use	6 (2.0%)	4 (2.6%)	2 (1.4%)	0.457
Antihypertensive use	122 (40.4%)	52 (33.3%)	70 (47.9%)	0.010**
Anti-diabetic use	37 (12.3%)	15 (9.6%)	22 (15.1%)	0.149
Antiplatelet use	59 (19.5%)	32 (20.5%)	27 (18.5%)	0.658
Statin use	33 (10.9%)	20 (12.8%)	13 (8.9%)	0.276
Baseline WMHs-ARWMC scores*	3 (0.00–7.00)	0 (0.00–3.00)	7 (4.00–10.00)	< 0.001**
Baseline PVWMHs-Fazekas scores				< 0.001**
None	124 (41.1%)	105 (67.3%)	19 (13.0%)	
Mild	92 (30.5%)	37 (23.7%)	55 (37.7%)	
Moderate	59 (19.5%)	11 (7.1%)	48 (32.9%)	
Severe	27 (8.9%)	3 (1.9%)	24(16.4%)	
Baseline SCWMHs-Fazekas scores				< 0.001**
None	138 (45.7%)	111 (71.2%)	27 (18.5%)	
Mild	83 (27.5%)	32 (20.5%)	51 (34.9%)	
Moderate	54 (17.9%)	9 (5.8%)	45 (28.8%)	
Severe	27 (8.9%)	4 (2.6%)	23 (15.8%)	
Baseline ICAS				< 0.001**
Grade 1	178 (58.9%)	123 (78.8%)	55 (37.7%)	
Grade 2	72 (23.8%)	23 (14.7%)	49 (33.6%)	
Grade 3	52 (17.2%)	10 (6.4%)	42 (28.8%)	

BMI, body mass index; BP, blood pressure; PVWMHs, periventricular white matter hyperintensities; SCWMHs, subcortical white matter hyperintensities; ICAS, intracranial atherosclerotic stenosis.

*Median (interquartile range), using Mann–Whitney *U* test.

**Significant variables.

ARWMC scores, Age-related White Matter Changes (ARWMC) rating scale scores; WMHs progression, increase in one point or more on the modified Rotterdam Progression scale (mRPS).

### Association between baseline ICAS severity and risk of WMHs progression

We used binary logistic regression analyses to calculate the ORs for ICAS severity with the progression of WMHs, as shown in Table 2. Compared to the patients with Grade 1 ICAS, those with Grade 2 ICAS had an OR of 4.8, and those with Grade 3 ICAS had an OR of 9.4 (unadjusted model in Table 2). The association remained statistically significant after adjusting for age and sex (model 1 in Table 2) with declined ORs [3.7 (95% CI 2.0–6.8) for Grade 2 ICAS; 6.4 (95% CI 2.9–14.2) for Grade 3 ICAS; *P* < 0.001, separately]. The effect magnitudes were changeless after additional adjustment for VRFs (model 2 in Table 2). It remained significant but with a slightly faded impact when further correcting for baseline WMHs (OR 2.8, 95% CI 1.4–5.5 for Grade 2 ICAS; OR 3.0, 95% CI 1.2–7.3 for Grade 3 ICAS; *P* < 0.001, separately; model 3 in Table 2). The correlation for age and baseline WMHs (ARWMC scores) with WMHs progression was weaker, but still significant (OR 1.1, 95% CI 1.0–1.1, *P* = 0.010; OR 1.3, 95% CI 1.2–1.4, *P* < 0.001; respectively).

**Table 2:** Logistic regression analysis of baseline ICAS severity with risk of WMHs progression.

	**Unadjusted Model**		**Adjusted OR (95% CI)**					
	**OR (95% CI)**	***p***	**Model 1**	***p***	**Model 2**	***p***	**Model 3**	***p***
Age	1.1 (1.1–1.2)	< 0.001	1.1 (1.1–1.1)	< 0.001	1.1 (1.1–1.1)	< 0.001	1.1 (1.0–1.1)	0.010
Hypertension	2.5 (1.5–4.0)	< 0.001	–	–	1.6 (0.9–2.8)	0.116	1.4 (0.8–2.6)	0.270
Diabetes	2.5 (1.3–4.9)	0.006	–	–	1.6 (0.7–3.4)	0.275	2.1 (0.9–5.0)	0.105
Previous stroke	2.7 (1.3–5.6)	0.007	–	–	1.6 (0.7–3.9)	0.254	0.9 (0.3–2.4)	0.818
Baseline WMHs*	1.4 (1.3–1.5)	< 0.001	–	–	–	–	1.3 (1.2–1.4)	< 0.001
**Baseline ICAS**								
Grade 1	1.0 (reference)		1.0 (reference)		1.0 (reference)		1.0 (reference)	
Grade 2	4.8 (2.6–8.6)	< 0.001	3.7 (2.0–6.8)	< 0.001	3.2 (1.7–6.2)	< 0.001	2.8 (1.4–5.5)	0.003
Grade 3	9.4 (4.4–20.1)	< 0.001	6.4 (2.9–14.2)	< 0.001	5.1 (2.1–12.1)	< 0.001	3.0 (1.2–7.3)	0.015
Ptrend	3.5 (2.4–5.0)	< 0.001	2.8 (1.9–4.1)	< 0.001	2.5 (1.6–3.7)	< 0.001	1.8 (1.1–2.8)	0.002

OR, odds ratio; CI, confidence interval; WMHs, white matter hyperintensities; ICAS, intracranial atherosclerotic stenosis.

Model 1, adjusted for age and sex; Model 2, additionally adjusted for vascular risk factors (e.g., hypertension, diabetes, coronary heart disease, dyslipidaemia, previous stroke, current smoking and daily alcohol use); Model 3: additionally adjusted for baseline WMHs.

*Baseline WMHs, measured by Age-related White Matter Changes (ARWMC) rating scale; WMHs progression, increase in one point or more on the modified Rotterdam Progression scale (mRPS).

### Effect of baseline ICAS severity on WMHs progression scores on MRPS

**Figure 2:**
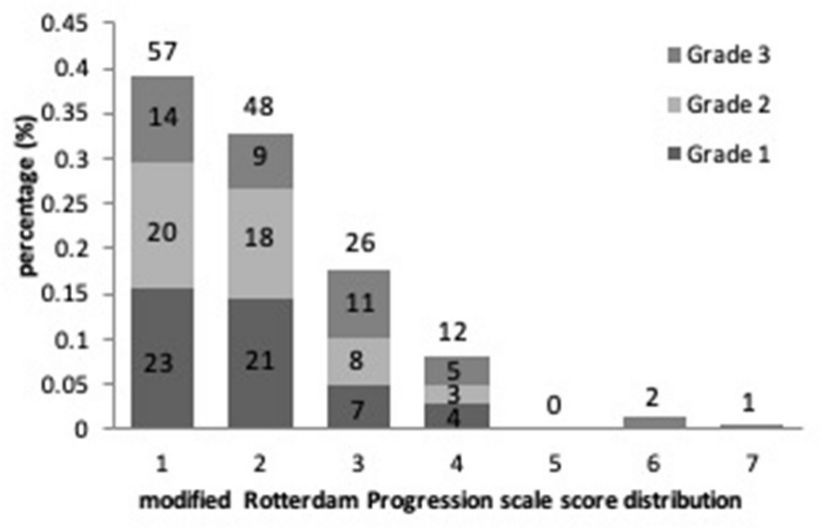
The proportional frequency of modified Rotterdam Progression scale (mRPS) scores and its distribution with ICAS severity are depicted.

Results of the linear regression analysis for ICAS degrees were described in Table 3. Patients with Grade 2 and 3 ICAS, in comparison with Grade 1 ICAS, gained higher increase on mRPS (Figure 2), and showed a significant influence on WMHs progression in univariate linear regression (*P* < 0.001). Therefore, we performed a multiple linear regression analysis and found that: ICAS severity was significantly associated with WMHs progression scores, higher for Grade 3 ICAS [β (SE) = 0.18 (0.18), *P* = 0.001] followed by Grade 2 ICAS [β (SE) = 0.10 (0.15), *P* = 0.044] compared with Grade 1 ICAS; and it remained notably significant in terms of baseline WMHs (ARWMC scores) relating to increase of mRPS scores [β (SE) = 0.53 (0.01), *P* < 0.001].

**Table 3:** Linear regression for the association between baseline ICAS severity and the WMHs progression scores.

	**Univariate linear regression**		**Multiple linear regression**	
	**β (SE)**	***p***	**β (SE)**	***p***
Age	0.30 (0.01)	< 0.001	0.06 (0.01)	0.202
Hypertension	0.23 (0.15)	< 0.001	0.05 (0.13)	0.342
Dyslipidaemia	−0.12 (0.20)	0.032	−0.01 (0.17)	0.767
Previous stroke	0.19 (0.22)	0.001	−0.00 (0.19)	0.981
Baseline WMHs*	0.61 (0.01)	< 0.001	0.53 (0.01)	< 0.001
**Baseline ICAS**
Grade 2 vs. Grade 1	0.13 (0.17)	< 0.001	0.10 (0.15)	0.044
Grade 3 vs. Grade 1	0.36 (0.18)	< 0.001	0.18 (0.18)	0.001

β, standardized regression coefficient; SE, standard error; WMHs, white matter hyperintensities; ICAS, intracranial atherosclerotic stenosis.

*Baseline WMHs, measured by Age-related White Matter Changes (ARWMC) rating scale.

WMHs progression scores, the modified Rotterdam Progression scale (mRPS) scores.

### Risk for WMHs progression subtypes associated with ICAS severity at baseline

**Figure 3:**
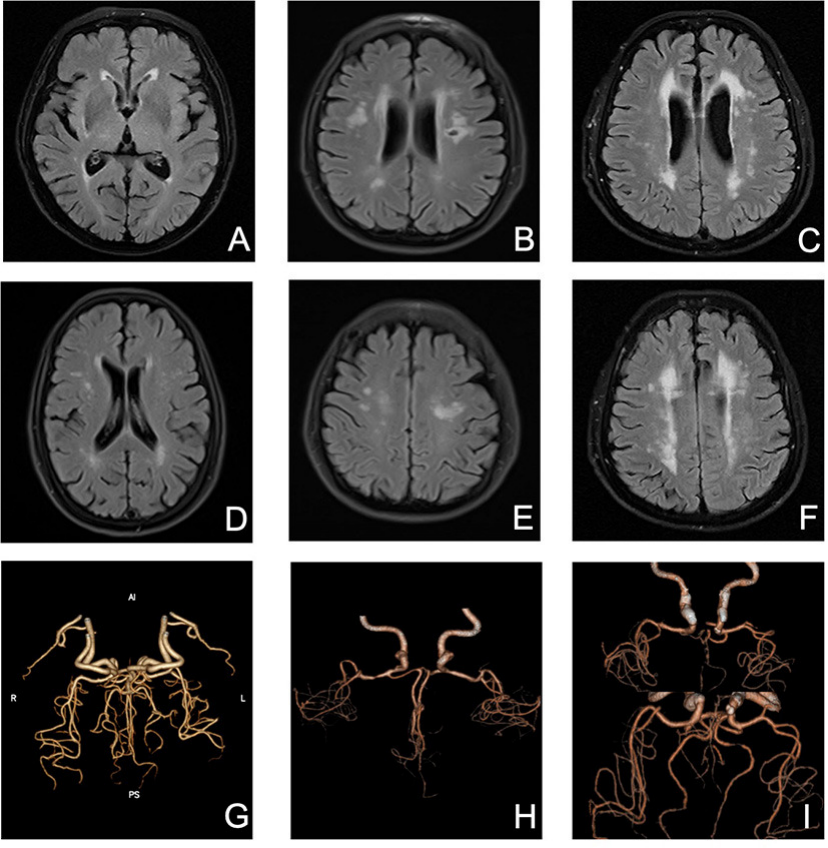
Several cases with different WMHs and ICAS. **(A)** mild PVWMHs; **(B)** moderate PVWMHs; **(C)** severe PVWMHs; **(D)** mild SCWMHs; **(E)** moderate SCWMHs; **(F)** severe SCWMHs; **(G)** Grade 1 ICAS; **(H)** Grade 2 ICAS; **(I)** Grade 3 ICAS.

Presence of WMHs progression was stratified separately into PVWMHs (*n* = 104), SCWMHs (*n* = 106), basal ganglia (*n* = 39), and infratentorial region (*n* = 5) progress according to mRPS (including coexistence of two or more regions). To search for a detail relationship of ICAS degree with WMHs progression subtypes, we repeated the logistic regression analysis with separate PVWMHs and SCWMHs progression in Table 4 and found that: after adjusting for confounding variables [age, sex, VRFs and baseline WMHs (Fazekas scores of PVWMHs and SCWMHs, respectively)], the ORs associated with Grade 2 and Grade 3 ICAS at baseline were 2.31 (95% CI 1.21–4.39, *P* = 0.011) and 2.50 (95% CI 1.18–5.32, *P* = 0.017) for PVWMHs progression, respectively, and remained significant but slightly weakened to 1.92 (95% CI 1.01–3.68, *P* = 0.048) and 2.47 (95% CI 1.14–5.34, *P* = 0.022) for SCWMHs progression, respectively. Figure 3 shows several cases with different WMHs and ICAS.

**Table 4:** Risk for progression of PVWMHs and SCWMHs associated with ICAS severity at baseline.

	**Risk for PVWMHs progression**				**Risk for SCWMHs progression**			
	**Unadjusted**		Adjusted*		**Unadjusted**		Adjusted*	
	**OR (95% CI)**	***p***	**OR (95% CI)**	***p***	**OR (95% CI)**	***p***	**OR (95% CI)**	***p***
Baseline PVWMHs	3.10 (2.30–4.17)	< 0.001	2.64 (1.94–3.62)	< 0.001	–	–	–	–
Baseline SCWMHs	–	–	–	–	3.44 (2.53–4.67)	< 0.001	3.08 (2.24–4.22)	< 0.001
**ICAS**
Grade 1	1.00 (reference)	–	1.00 (reference)		1.00 (reference)		1.00 (reference)	
Grade 2	3.81 (2.12–2.90)	< 0.001	2.31 (1.21–4.39)	0.011	2.83 (1.58–5.05)	< 0.001	1.92 (1.01–3.68)	0.048
Grade 3	5.63 (2.90–10.9)	< 0.001	2.50 (1.18–5.32)	0.017	5.35 (2.77–10.3)	< 0.001	2.47 (1.14–5.34)	0.022

PVWMHs, periventricular white matter hyperintensities; SCWMHs, subcortical white matter hyperintensities; ICAS, intracranial atherosclerotic stenosis.

*Adjusted for age, sex, vascular risk factors (e.g., hypertension, diabetes, coronary heart disease, dyslipidaemia, previous stroke, current smoking and daily alcohol use) and baseline PVWMHs and SCWMHs (measured by Fazekas scores), respectively.

Progression for PVWMHs and SCWMHs, increase in one point or more on the PVWMHs and SCWMHs part of modified Rotterdam Progression sc scale (mRPS), respectively.

## Discussion

Although the pathophysiology of WMHs remains hypothetical, there is evidence that WMHs may have correlation with cerebral ischemia. Chronic hypoperfusion of brain parenchyma could render white matter in loss of myelin and axons and tissue rarefaction (24), somewhat analogous to pathological manifestations of WMHs. Reduced cerebral blood flow was found in white matter instead of gray matter among individuals with WMHs and the local circulatory deficiency was related to WMHs volume of the same lobe (25). White matter is particularly vulnerable to hypotension-induced low flow effects because it is supplied by long, non-anastomosing arterioles with deficient autoregulatory capacity. Intracranial arteries, as the vessel most neighboring to upstream, play a critical role in distal perfusion into cerebral small vessels and parenchyma, and exhibit distinct metabolism and relatively blunted autoregulation compared with extracranial arteries for lacking vasa vasorum and external elastic lamina (26). Hence, there have been reasons to believe that ICAS plays a role in the pathogenesis and deterioration of WMHs, which is the purpose of our study.

In our analysis, the progression of WMHs measured by mRPS occurred in 48.3% of Chinese patients and could be trebled by Grade 3 ICAS in 3 years. Remarkably, a dose-response relationship between ICAS degrees and mRPS scores was found additionally in a linear regression, which undoubtedly adds weight to the strong impact of ICAS on WMHs burden increase. In previous cross-sectional studies, greater prevalence of WMHs were elucidated in ICAS rather than ECAS (11) and the former could act as an independent factor associated with greater WMH burden in Korean patients (12), while these were not in agreement with another study in American crowd (14). As a fact, those studies took acute stroke patients as research objects and had no age-matched group with stroke-free participants, which may cause indeterminate validity and inevitably bias for the association. A recent Chinese cohort study found that WMHs were prone to be hemodynamically compromised by the impact of ICAS, but it did not consider the influence of isolated or concurrent extracranial stenosis (7). Regrettably, no study on WMHs progression has been performed so far. Thus, our results provide new insights into understanding the mechanisms underlying WMHs recruitment and the likelihood of ICAS in determining the severity of WMHs progression in ECAS free patients.

We found that more severe ICAS remained relevant with PVWMHs progression but was with an attenuated relation to the SCWMH progression even after adjusting for confounding variables. Periventricular white matter is prone to be more vulnerable to hemodynamic disturbance than subcortical white matter, since this area is supplied by non-collateralizing subependymal arteries with unstable blood supply. However, previous cross-sectional studies showed that SCWMHs gained more influence by chronic hypoperfusion secondary to atherosclerotic stenosis than PVWMHs (12, 27), which were not in complete agreement with our study. Moreover, Sachdev et al. (28) also discovered that the deep white matter and anterior brain regions performed a greater rate of progression. Interestingly, as a notable result in our analysis, baseline SCWMHs themselves manifested a stronger power on WMHs progression than PVWMHs, which corresponds with the results reported by Umemura et al. (29) that higher mRPS scores were found in SCWMHs with relation to baseline SCWMHs severity. These may suggest that PVWMHs and SCWMHs have dissimilar pathogenic mechanisms as the burden of lesions increases.

There are limited longitudinal studies investigating the severity of ICAS in elderly patients with relation to WMHs progression. We used CTA as a non-invasive examination method for the diagnosis and assessment of ICAS to enhance methodological effectiveness. In addition, we categorized WMHs progression into PVWMHs and SCWMHs and found the potential distinctive features between them. ECAS (with a stenosis of ≥20%) was eliminated in our study due to the controversial relationship between carotid stenosis/plaque and WMHs (30) and to avoid confounding interfere with blood flow.

Several limitations to the present study should be addressed. First, as our study retrospectively utilized inpatients from a single center who sought medical service for various reasons, it may be insufficiently representative of community-based cohorts who appear to have fewer risk factors. Nevertheless, the longitudinal data and blinded assessment may strengthen its reliability. An extrapolation to other ethnic individuals could be tested in future studies. Second, although automated assessment of WMHs burden was preferred over a visual scales in single center studies, visual rating scales have proven to be reliable and showed good correlation with volumetrics and interobserver agreement for WMHs assessment (23). We also expect that computerized appraisal procedure would be available in our future studies. Third, a much lower prevalence of ICAS is presented in our study, mainly the Grade 2 ICAS with a prevalence of 23.8% than that of 42.0% reported by Dolan et al. (20) by postmortem. However, the participants who had completed body autopsies had a mean age of 87.6 years at death which was definitely older than that in our study, and the subjects with ECAS and acute stroke who share more risk factors were eliminated from our analysis.

In summary, our results demonstrate the association of ICAS severity to WMHs progression in elderly Chinese patients and additionally have distinct relevancy to PVWMHs and SCWMHs, which may provide clues for understanding atherosclerotic stenosis of relatively large intracranial vessels as an indicator of widespread microvascular lesions and microcirculation defects not only potential responsible vessels resulting in deficit cerebral perfusion. Further studies with a larger sample size are needed to confirm the conclusions and to investigate the role of ICAS in prevention of WMHs progression.

## Data availability statement

The raw data supporting the conclusions of this article will be made available by the authors, without undue reservation.

## Ethics statement

The studies involving human participants were reviewed and approved by the Institutional Review Board of the Third Military Medical University. The patients/participants provided their written informed consent to participate in this study.

## Author contributions

All authors contributed to the article and approved the submitted version.

## Funding

This work was supported by the Construction Project of Capacity Improvement Plan for Chongqing Municipal Health Commission Affiliated Unit (2019NLTS001)-ZS03174, the Operating Grant to Chongqing Key Laboratory of Neurodegenerative Diseases (1000013), Chongqing Talent Project (2000062), Overseas Students Entrepreneurial Fund (2000079), and Plan for High-level Talent Introduction (2000055).

## Conflict of interest

The authors declare that the research was conducted in the absence of any commercial or financial relationships that could be construed as a potential conflict of interest.

## Publisher's note

All claims expressed in this article are solely those of the authors and do not necessarily represent those of their affiliated organizations, or those of the publisher, the editors and the reviewers. Any product that may be evaluated in this article, or claim that may be made by its manufacturer, is not guaranteed or endorsed by the publisher.
